# Influence of platinum harmonized textile on neuromuscular, systemic and subjective recovery

**DOI:** 10.1371/journal.pone.0186162

**Published:** 2017-10-12

**Authors:** Fridolin Zinke, Patrick Bakenecker, Daniel Hahn

**Affiliations:** 1 Human Movement Science, Faculty of Sport Science, Ruhr-University Bochum, Bochum, Germany; 2 School of Human Movement and Nutrition Sciences, University of Queensland, Brisbane, Australia; Universite de Nantes, FRANCE

## Abstract

The clothing manufacturer Venex Life-Science tracks the strategy to enhance recovery process through implementing platinum harmonized technology (PHT) into their Venex^®^ Regeneration Wear. The proposed mechanisms of the regeneration wear are an increase of parasympathetic activity and an increase of the temperature due far-infrared radiation. The purpose of this study was to investigate potential effects of Venex regeneration wear on the recovery of neuromuscular, systemic and subjective parameters following exercise. In a randomized, single-blinded cross-over design, 13 strength-trained male participants performed two exercise sessions in one day. After the first exercise session and for 3 nights following the exercise day participants wore the Venex regeneration wear or a placebo product. Measures of isometric maximum voluntary contraction (MVC), voluntary activation (VA) and potentiated twitch torque (PTT) of the knee extensors, as well as maximum jump height during the Counter-Movement and Drop Jump, creatinkinase (CK) and subjective parameters (perceived pain, recovery condition and sleep quality) were obtained before and after each exercise session and 24, 48, and 72 hours after the first exercise session. MVC, PTT, CK and jump parameters changed over time during the exercise day as well as on the following days, however, there were no significant differences between treatments. Subjective parameters showed strong effect sizes (i. e. Cohen’s *d* > 0.6) in favor for Venex but no significant differences between treatments. Based on these findings, it is concluded that wearing a platinum harmonized textile following exercise did not improve neuromuscular and systemic recovery in a trained male population to a statistical noticeable extent. However, the findings for the subjective parameters suggest some potential for enhanced recovery that requires further research.

## Introduction

To achieve the supreme goal of winning a competition, championship, world cup or Olympic Games event, peak performance is required. However, to achieve peak athletic performance and to stay free of injury, both the training regimes and recovery strategies between training sessions as well as overnight have to be well managed. Intense exercise or competitive events can lead after only a few minutes to fatigue which can occur at different levels of the organism (peripherally or centrally) and is shown for example by a decreased maximal force or a reduced rate of force development [1–3]. Additionally intense exercise may lead to dehydration and depletion of glycogen-stores [4,5] delayed muscle soreness and inflammation [6]. Accordingly, the recovery strategy should fit the demands of the training or competition, with the consequence to minimize, or ideally eliminate the initiated fatigue.

In this context, various recovery strategies are preferred by different athletes so that they can achieve an appropriate balance between training, competition and recovery to minimize or eliminate fatigue. A common goal of most recovery strategies is to increase blood flow in order to increase nutrient influx into the demanded system and to regulate inflammatory processes triggered by the training stimulus. Massages before and especially after strenuous exercise [7], active recovery [8], cold-water immersion as a form of cryotherapy [9] and compression garments [10] are conventional recovery strategies that are used to achieve increased blood flow. These same recovery strategies may also reduce muscle tension and depress neuromuscular excitability (e.g. massages [7]), reduce post exercise inflammation (e.g. cryotherapy [11]) or limit muscle swelling (e.g. compressive garments [10].

Improved recovery following training or competition is important because it allows to set training stimuli in a higher density (e.g. more training sessions per week), volume and/or intensity. However, there is debate regarding the mentioned strategies because of the limited and unambiguous evidence that the mentioned strategies actually improve the recovery process [12]. In most cases, personal preferences and external factors, such as the space and time available for recovery treatments, determine which recovery strategies are used.

A completely new approach to enhance recovery following training is the use of a non-compressive textile that contain fibers with platinum harmonized technology. These fibers are manufactured by VENEX Life Science and harmonized with nano-sized diamond (ND) and platinum-colloid (NP) (DPV576) [13,14] and implemented into a textile (DPV576-C) known as Venex^®^ Regeneration Wear. The fiber is manufactured through a method of attaching a dispersion of nano-sized diamond and platinum nanocolloids to the fibers or by a method of mixing it into the spinning solution. Importantly, the Venex garment is no compression wear but is worn as loose fitting pants and/or long sleeves or T-shirts. The recovery effect of this textile is supposed to be mediated through a transient receptor potential vanilloid (TRPV) located in the skin. Through this receptor, DPV576 activates keratinocytes, which downregulates the ion channel transient receptor potential vanilloid 4 (TRPV4) and an increases the secretion of nerve growth factor (NGF) [15]. Vanilloid-like channels are linked with circadian body temperature regulation and the autonomous nervous system [16], whereas NGF plays a major role in the regulation of circadian rhythms [17]. The second effect of DPV576-C is a temperature-regulating effect, which is caused by the far-infrared radiation of the ND and NP [18]. Based on these findings the manufacturer postulates that their regeneration wear, which contains the platinum harmonized fabric (DPV576-C), will increase blood flow and activate the parasympathetic nervous system, and as a result of this, improving the recovery process. Additionally, the nano-material (DPV576) has been shown to stimulate antigen-presenting cells and lymphocytes [19]. Dendritic cells that are located in the skin are activated by DPV576 for a more efficient T-cell proliferation and maturation [20]. Specifically, a solution of ND and NP increased the percentages of T-lymphocytes CD4^+^ and CD8^+^ of aged mice, which may be useful for preventing immune dysfunction [21]. Previous studies that have investigated the effects of DPV576 on cancer cells have shown that this solution of ND and NP is an effective tool for fighting cancer cells [22–24]. Although the toxicity of nanomaterial is dose- and time-dependent [25], we are not aware of any published studies that have shown the nanomaterial used in the Venex textile is toxic to humans or animals. It has been shown that NDs are non-toxic to a diversity of cell types [26], however the overall effects of nanomaterials are not completely known [19,25].

Concerning the manufacturer’s proposed effects of their regeneration wear, we are only aware of one study that has investigated the influence of Venex wear on parasympathetic activity. This study was carried out with soccer athletes which were time-tested in a curved sprint and short dribble test following two training sessions on one day. Time in the curved sprint and heart rate variability indicated an improvement in favor of the experimental group that wore the PHT in between sessions [27]. Further, this study found that wearing a textile containing PHT led to increased values of pNN50 and r-MSSD, which are parameters measured through heart rate-variability and suggest increased parasympathetic activity [28].

Therefore and from an applied perspective, the purpose of this study was to investigate the effects of the Venex regeneration wear on the recovery of neuromuscular, systemic and subjective parameters following two exercise sessions on the same day in a randomized, single-blinded cross-over design. The goal of this study was not to determine at which level the proposed effects were occurring nor to verify the various mechanisms of the platinum harmonized technology, but instead to determine if there are any neuromuscular, systemic or subjective changes following training that results from wearing the textile. Based on the proposed mechanisms of DPV576-C, we hypothesized that participants would experience a faster recovery of neuromuscular, systemic and subjective parameters following application of the Venex textile compared to a placebo product. We also postulated that that the neuromuscular, systemic and subjective parameters would decrease less between the two exercise sessions as well as after the second exercise session when participants wore the Venex textile.

## Methods

### Participants

Fourteen healthy and strength-trained male participants were recruited and provided written informed consent prior to participating in the study. One participant was forced to drop out due to dental surgery so that the results are based on thirteen participants (age 24.4 ± 2.6 years, minimum 21 years, maximum 29 years; height 182.1 ± 9.2 cm; body mass 79.7 ± 6.0 kg; 10 repetition maximum (RM) in the back squat 91.7 ± 14.0 kg). For the recruitment of participants flyers have been published in the Faculty of Sport Science at Ruhr-University Bochum two months prior the start of the study (November 2015). Participants were free of any kind of injury and were required to squat their body weight as additional load in a Smith machine (Heinz Kettler GmbH & Co. KG, Ense-Parsit, GER) at least ten times as an inclusion criteria. All experimental procedures were approved by the Ethics Committee of the Faculty of Medicine at Ruhr-University Bochum and all procedures were conducted in accordance with the Declaration of Helsinki. The data will be available indefinitely on request without compromising the subjects’ privacy.

### Study design

The aim of the study was to investigate potential effects of a new regeneration wear compared to a placebo product in a randomized, single-blinded cross-over design. Participants therefore received both treatments (Venex (V) and placebo (P)) in a randomized order and were unaware of which product they were given because the color and feel of the textiles were identical as they were both manufactured by Venex. Further, the Venex and placebo products were unlabeled so that participants could not identify the brand of the regeneration wear. The washout-phase between the treatments was at least six weeks to ensure that the Venex regeneration wear did not have an influence on the physiological, neuromuscular and subjective parameters in the subsequent testing session [29,30]. To avoid any placebo effects, participants were informed that the study’s aim was to investigate the effects of two different types of regeneration wear. They were instructed to avoid any kind of physical exercise or competition two days prior the exercise day and during the three following days. Importantly, the clothing-textile induced no compression on the skin when it was worn and the sizes of the pants and shirt were both selected for a loose and comfortable fitting.

### Protocol

**Fig 1:**
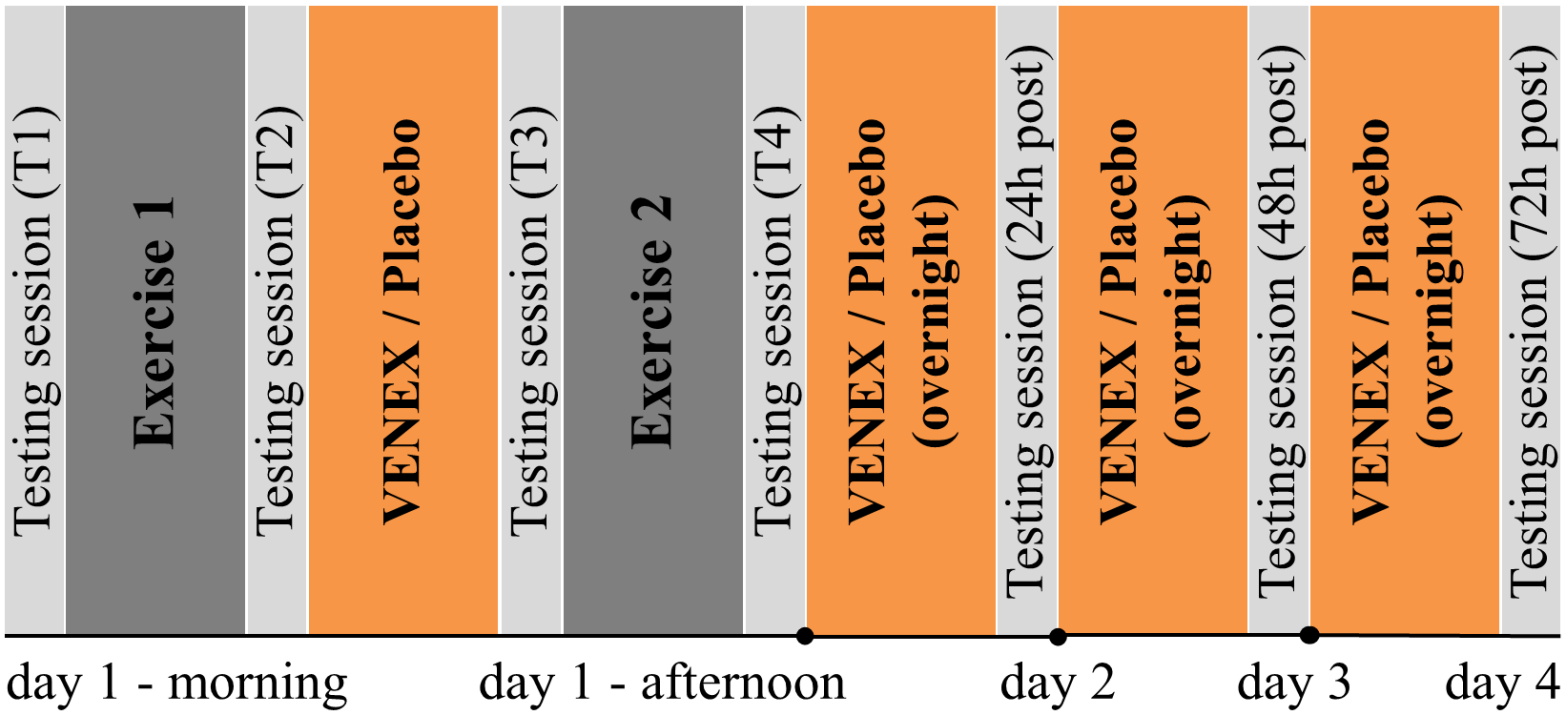
Study protocol. The testing and exercise procedure consisted of 7 testing sessions and two exercise session. Day 1 started with baseline testing (T1) prior to the first exercise, which was followed by the second testing (T2). After T2 subjects had to rest for three hours wearing Venex or Placebo during the first hour of rest. Afterwards, the third testing (T3) was performed just before the second exercise and a subsequent fourth testing (T4). Following the exercise day, participants had to wear either Venex or placebo overnight. Testing sessions 5, 6, and 7 took place 24h, 48h and 72 hours after T1. This protocol was executed twice in a single-blinded cross-over design so that every participant run through the process once with the Venex wear and once with the placebo product.

Participants performed two exercise sessions with four testing sessions in one day and post-testing sessions for the following three days for both treatments. After a standardized warm-up, the first baseline testing session (T1) was carried out prior to the first exercise session and the second testing session (T2) was performed immediately after the first exercise session. Then, participants rested for three hours and subsequently performed the third testing session (T3). Immediately following this, the second exercise session and fourth testing session (T4) was performed. Post-testing sessions T5, T6 and T7 took place 24h, 48h, and 72 hours following the first testing session, respectively (Fig 1). After the first exercise session, participants wore P and V long sleeves and pants for one hour between the exercise sessions and during the three nights after the exercise day. During the three hours of rest between the exercise sessions, participants laid in a supine position for the first hour when wearing V or P, but were not allowed to nap. Afterwards, participants were allowed to move freely in the facility where all testing and exercise sessions were carried out. This procedure was used to simulate a typical training day of an athlete, who would probably rest after training, but has to deal with everyday tasks between training sessions. At least 72h before, but not more than 1 week prior to the first testing session, participants attended a familiarization session, where they performed a 10RM test of the squat exercise and became familiar with the warm-up procedure and testing session. The testing measures were performed in a systematic order as follows: Capillary blood sampling, questionnaires, jump testing and dynamometer testing (which included the interpolated twitch technique [31]). The warm-up procedure involved 7 minutes of ergometer-cycling at 100 Watts, followed by different running drills and dynamic stretching of the lower extremity. This warm-up procedure was performed before every testing session except the testing sessions immediately after the exercise sessions. For the 10RM testing, participants performed two warm-up sets with six to eight repetitions with an individually selected submaximal load. Afterwards, participants had to estimate their 10RM. If the participants noticed after six repetitions that the load was too light they stopped the first set and increased the load by 5kg. Following a five-minute rest, participants performed the next set. The procedure was repeated until the participant was not able to perform more than 10 repetitions with the additional load.

Nutrition is a major factor for recovery, so we attempted to keep the diets of the participants consistent for the duration of the testing sessions by having participants document their diet on the exercise day and the three days following. One day prior to the second treatment the nutrition logs were handed back to the participants and they were encouraged to follow the same diet as they did during their first treatment. Because all participants were sport students and the whole study was performed during the semester (no practical or theoretical exams during that time) there were no changes in physical activity of the participants between the two treatments.

### Exercise

To simulate a training day that is typical for most competitive sports, the participants completed two exercise sessions in one day. In the morning, participants performed a power exercise, which included five sets of 20 unilateral alternating Step-Up Jumps (10 jumps per leg) with a one-minute rest between sets. After a three-minute rest, participants then performed ten sets of ten repetitions of Countermovement-Jumps (CMJs), with one-minute rest between sets. The goal of the CMJ was to jump as high as possible after lowering the body until the thighs were parallel to the ground. Whether the thigh was parallel to the ground was visually controlled by the investigator and the participants received verbal feedback after every jump. The second exercise session in the afternoon involved a high volume strength exercise. Participants had to perform ten sets of ten squats, with a cadence of two seconds in the lowering and lifting phases. Participants had to lower their body until their thighs were parallel to the floor, which was standardized via an electric position-marker that gave an acoustic signal when the appropriate depth was reached. The additional load for each participant in the first set was their 10RM. If participants were not able to perform all ten repetitions or needed more than 40 seconds to complete one set of squats, the additional load was decreased by 5kg. The rest in between sets was two minutes.

### Neuromuscular performance testing

An isokinetic dynamometer (IsoMed2000, D&R Ferstl, GmbH, Hemau, GER) was used to measure isometric knee extension torques during maximum voluntary isometric contractions (MVC) of the knee extensors. Prior to testing, the right knee was positioned at 60° of knee flexion (0° refers to straight leg) and shoulder and waist straps were used to secure participants firmly in the seat of the dynamometer. The hip angle was fixed at 90° (thigh relative to torso). Maximal verbal encouragement was given by the investigator. During the MVC, the femoral nerve was electrically stimulated with a high-voltage stimulator (DS7AH, Digitimer Stimulator Ltd., UK) to produce an involuntary superimposed twitch (ST) to estimate voluntary activation. Three seconds after the MVC, a potentiated twitch (PT) was given to estimate voluntary activation (VA) and peripheral fatigue. VA was calculated from the relationship of the superimposed twitch torque (STT) and potentiated twitch torque (PTT), whereas changes in the PTT by itself can inform about peripheral fatigue in the tested muscle. For nerve stimulation, the femoral nerve was localized by stimulating different spots in the groin region with a motor point pen (Compex Medical SA—Switzerland) at 50 mA (1ms pulse width). The stimulating cathode (ValuTrode Clthrnd. Axelgaard Manufacturing 3.2 cm diameter) was finally placed at the site where the greatest peak-to-peak knee extension torque occurred after stimulation and the anode was placed on the iliac crest. Then the stimulator current (1ms rectangular pulse) was progressively increased in 20mA steps from 100mA with 30 seconds of rest between stimulations, until no further increase in twitch torque was seen [32]. The stimulator current at maximum torque was then multiplied by a factor of 1.3 to ensure supramaximal motor nerve stimulation. All MVCs were carried out three times during each testing session and four minutes of rest was provided between MVCs. The stimulus during MVC was delivered on the plateau of the torque-time trace and an additional stimulus was delivered three seconds after the contraction, which resulted in a potentiated twitch.

Torque data was sampled at 1 kHz and synchronized using a 16-bit Power1401 and Spike2 data collection software (Cambridge Electronic Design, UK) and smoothed using a 10 ms moving average routine before further analysis. MVC was determined as peak to peak torque between baseline, when participants were in a completely relaxed state, and maximum torque during the voluntary contraction, before the superimposed twitch was induced. STT was calculated as the torque difference between the torque at the instant of the stimulation and the peak torque after the stimulation. PTT amplitude was determined as peak to peak torque between baseline after the MVC and maximum torque during the PT. VA was calculated with the formula: VA = (1-STT/PTT) x 100 [33]. VA was analysed for twelve participants only because data from one participant was not evaluable. For MVC and PT, the trial with the greatest peak-to-peak torque output was chosen for further analysis.

#### Jump parameters

Participants performed three Counter-Movement Jumps (CMJs) followed by three Drop Jumps (DJs) on a contact platform (Sportservice Foss, GER), with jumps separated by at least 20 seconds. During both jumps, participants placed their hands on their hips. For the DJs, participants had to drop from a box that was 40cm in height, and upon landing jump as high as possible with the shortest ground contact time possible. Flight-time and contact-time were measured for all jumps. Jump height for CMJ and DJ was calculated via flight time through the Haynl Tapping Software (Version 2.5.0.30). For DJs, the reactive-strength performance (RSP) was calculated via the formula RSP = Height[cm] / Contact time[s] [34], which has also been referred to as the reactive-strength index [35]. The CMJ with the highest jump height and the DJ with the best RSP was used for further analysis.

### Creatinkinase

For the measurement of creatinkinase-activity, 200 μl blood was sampled from the earlobe. Samples were collected in a serum gel tube with clotting activator (KABE Labortechnik GmbH, Nümbrecht-Elsenroth, Germany). 30 minutes following blood collection, the sample was centrifuged at 3800r/min (Heraeus COMBIFUGE) and stored at -20°C, until samples were analyzed via an INTEGRA gauge (Roche Diagnostics, Grenzach-Wyhlen, GER). CK activity from one participant was inexplicably high during one treatment it would have biased the results, so it was analyzed qualitatively only.

### Questionnaire

Participants had to specify their subjective pain intensity on a numeric horizontal scale from 1 (no pain) to 10 (worst pain) during each testing session [36]. Similarly, overall recovery condition and sleep quality were tracked on a scale from 1 (worst recovery condition/sleep quality) to 10 (best recovery condition/sleep quality). Sleep duration was also specified by the participants with quarter hour increments (S1 File).

### Statistical analysis

All data were tested and confirmed for normality with the Kolmogorov-Smirnov-Test. A two-way (time, treatment) repeated measure analysis of variance (ANOVA) was used to determine differences among MVC, VA, PTT, CMJ, RSP and CK between testing sessions as well as between treatments. Bonferroni adjusted post-hoc tests were used if appropriate. For pain intensity, recovery condition, sleep quality and sleep duration differences over time and between treatments were tested using Friedman tests with post-hoc Wilcoxon tests if appropriate. Additionally, effect sizes between treatments (V versus P) were calculated as Cohen’s *d* [37]. Statistical significance was set at P < 0.05. All statistical analyses were performed by using SPSS (Version 22, SPSS Inc., Chicago, IL, USA).

## Results

### Exercise

Strength exercise showed no differences for time under tension (TUT), repetitions or load. TUT was 34.0 ± 1.2 seconds for Venex and 34.0 ± 1.7 seconds for placebo. During that time participants performed 9.8 ± 0.7 repetitions (V) and 9.8 ± 0.5 repetitions (P) with an additional load of 83.0 ± 13.9 kg (V) and 82.8 ± 13.8 kg for (P), respectively. The two treatments were 7 ± 1 weeks (50 ± 8 days) apart.

### Neuromuscular performance

**Fig 2:**
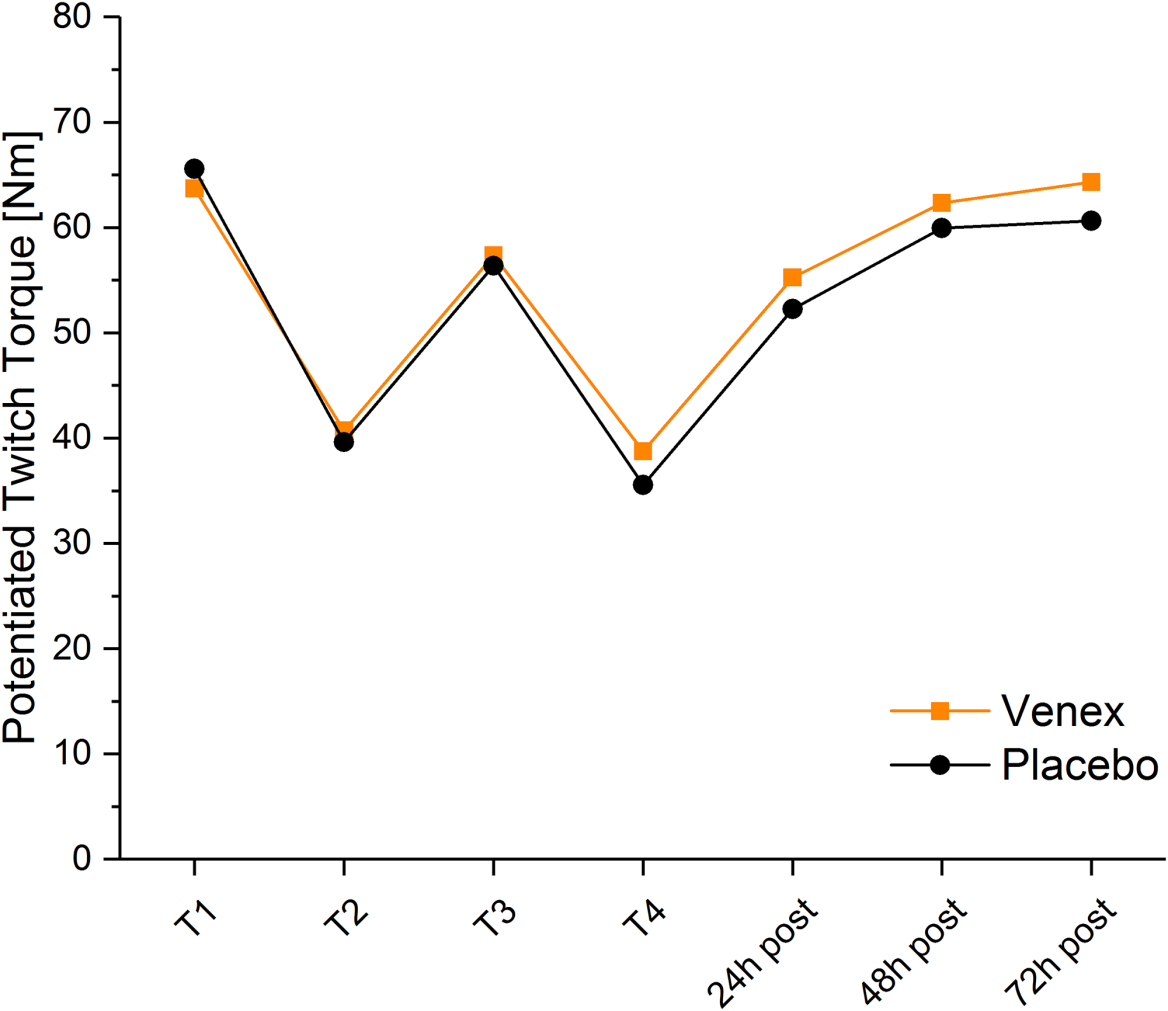
Potentiated twitch torque for both treatments over time. Data represent the mean ± 95% confidence intervals of the potentiated twitch torques.

**Fig 3:**
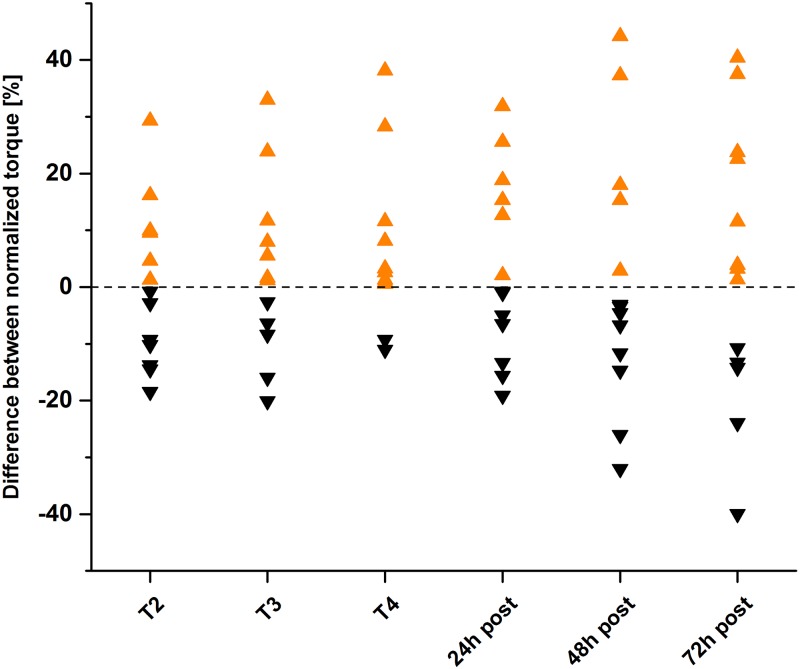
Individual normalized torque differences between baseline MVC measurement and post measurements. Difference between normalized torque for every participant at every testing session. Torque difference was calculated as the difference from normalized MVC Venex minus placebo. Orange symbols show a difference in favour for Venex and black symbols mean a difference in favour for Placebo. If a participant had a relatively higher MVC during the Venex treatment compared to the placebo treatment at a given testing session the value is an orange triangle.

For MVC and potentiated twitch baseline showed no significant differences between treatments. Overall, no significant difference between treatments but a significant main effect for time (p < 0.001) was found for MVC and potentiated twitch torque (PTT). For voluntary activation (VA) ANOVA indicated neither a systematic response to the exercise stimulus nor to the treatment with the Venex or placebo textile. All detailed information (mean and SD) is shown in Table 1. After the exercise sessions MVC torque was significantly decreased (T2), recovered partly during the resting period (T3) and dropped further after the second exercise session (T4). After the second exercise session MVC returned to baseline 48h after the exercise day for both treatments. The time course for PTT was similar to MVC. After a drop due to the first exercise session the PTT recovered during the recovery phase and was again significantly reduced after the second exercise session. 48 hours after baseline, PTT recovered to baseline (no significant difference to T1) again (Fig 2). Individual differences between normalized torque of both treatments are shown in Fig 3.

**Table 1:** Mean ± SD data from neuromuscular tests, jump performance, the biochemical marker and subjective parameters.

		T1	T2	T3	T4	24h post	48h post	72h post
*Neuromuscular performance*								
MVC (Nm)	Venex	284.0±68.9	233.6±59.9*	261.7±60.3	222.5±56.7*	248.5±51.5*	272.5±68.2	295.8±75.1
	Placebo	289.5±60.5	234.8±40.7*	260.9±47.2	202.3±41.9*	243.7±44.7*	270.6±46.0	287.5±46.2
Twitch (Nm)	Venex	63.7±14.9	40.7±12.7*	57.4±15.2	38.7±9.4*	55.3±11.7*	62.3±15.5	64.3±14.4
	Placebo	65.6±12.0	39.6±9.2*	56.4±11.0	35.6±10.3*	52.3±12.2*	59.9±11.9	60.6±12.4
VA (%)	Venex	88.4±7.8	87.7±10.9	87.6±9.6	88.5±4.8	88.9±7.0	90.9±6.2	92.6±6.1
	Placebo	88.9±9.7	89.5±10.2	88.0±6.5	86.2±8.6	91.2±5.6	86.5±8.3	91.9±5.6
*Jump performance*								
CMJ (cm)	Venex	43.7±7.2	37.9±6.5*	42.6±6.8	35.9±6.1*	40.4±6.5	41.9±5.5	42.5±6.6
	Placebo	43.3±8.0	36.5±8.6*	42.7±7.5	34.3±6.6*	40.4±5.9	41.1±6.8	43.6±6.8
RSP (cm/s)	Venex	176±45	138±37*	171±37	128±32*	151±38*	157±45	161±41
	Placebo	172±42	133±38*	174±50	119±.21*	150±.38*	153±39	165±39
*Biochemical marker*								
CK (U·L^-1^)	Venex	289±166	303±165*	350±164*	436±201*	679±397*	429±231	343±162
	Placebo	335±230	366±232*	383±202*	472±207*	641±311*	453±226	317±227
*Subjective parameters*								
PI	Venex	2.8±1.5	5.3±2.3*	4.0±1.4	6.2±2.0*	5.6±1.5*	6.2±1.8*	4.2±1.5
	Placebo	2.9±1.0	5.8±2.3*	3.8±1.4	6.2±1.6*	6.8±1.5*	7.1±2.1*	4.8±1.8
RC	Venex	7.8±1.5	4.3±1.8*	5.9±1.3*	2.8±1.4*	4.2±2.1*	4.6±1.9*	6.6±1.2
	Placebo	7.6±1.3	3.9±1.4*	6.1±1.7	2.5±1.1*	3.5±2.1*	3.5±1.9*	5.6±1.8
SQ	Venex					7.5±2.3	7.5±1.8	8.0±1.2
	Placebo					7.8±1.9	6.8±2.3	6.9±1.6

Parameters are shown as mean ± SD. T1: Test 1; T2: Test 2; T3: Test 3; T4: Test 4; MVC: Maximum voluntary contraction; VA: Voluntary activation CMJ: Counter-Movement Jump; RSP: Reactive-strength performance; CK: Creatinkinase activity; PI: Pain Intensity; RC: Recovery Condition; SQ: Sleep Quality;

* Significant difference from baseline measurement T1.

### Jump performance

No significant differences were found for the baseline measures of CMJ and RSP. Overall ANOVA indicated no significant difference between treatments but a significant difference for measurements over time for CMJ and RSP (Table 1). Time courses of jump performance were similar to MVC and PTT and are shown in Table 1. After the three hours rest between the two exercise sessions and 24h after the exercise day all jump parameters went back to baseline (no significant difference to T1).

### Creatinkinase activity

All samples were free of increased haemolysis that could have led to defective samples. Baseline measures showed no significant difference. ANOVA indicated no significant difference between treatments but a significant difference for measurements over time. CK activity showed a significant increase over time at T2, T3, T4 and a peak 24h post with an incline of 253 ± 158% with V and 244 ± 178% with P (p < 0.05) before it decreased to baseline at 48h post (Table 1). The qualitative analysis of the subject with an extraordinary increase in CK activity during the placebo treatment showed an incline from 109 (T1) to 1758 (24h post), 5382 (48h post) and >20000 U·L^-1^ (72h post). For Venex this subject’s CK activity increased from baseline 263 to 317 (24h post), 206 (48h post) and 163 U·L^-1^ (72h post).

### Perceived pain, recovery condition and sleep quality

**Fig 4:**
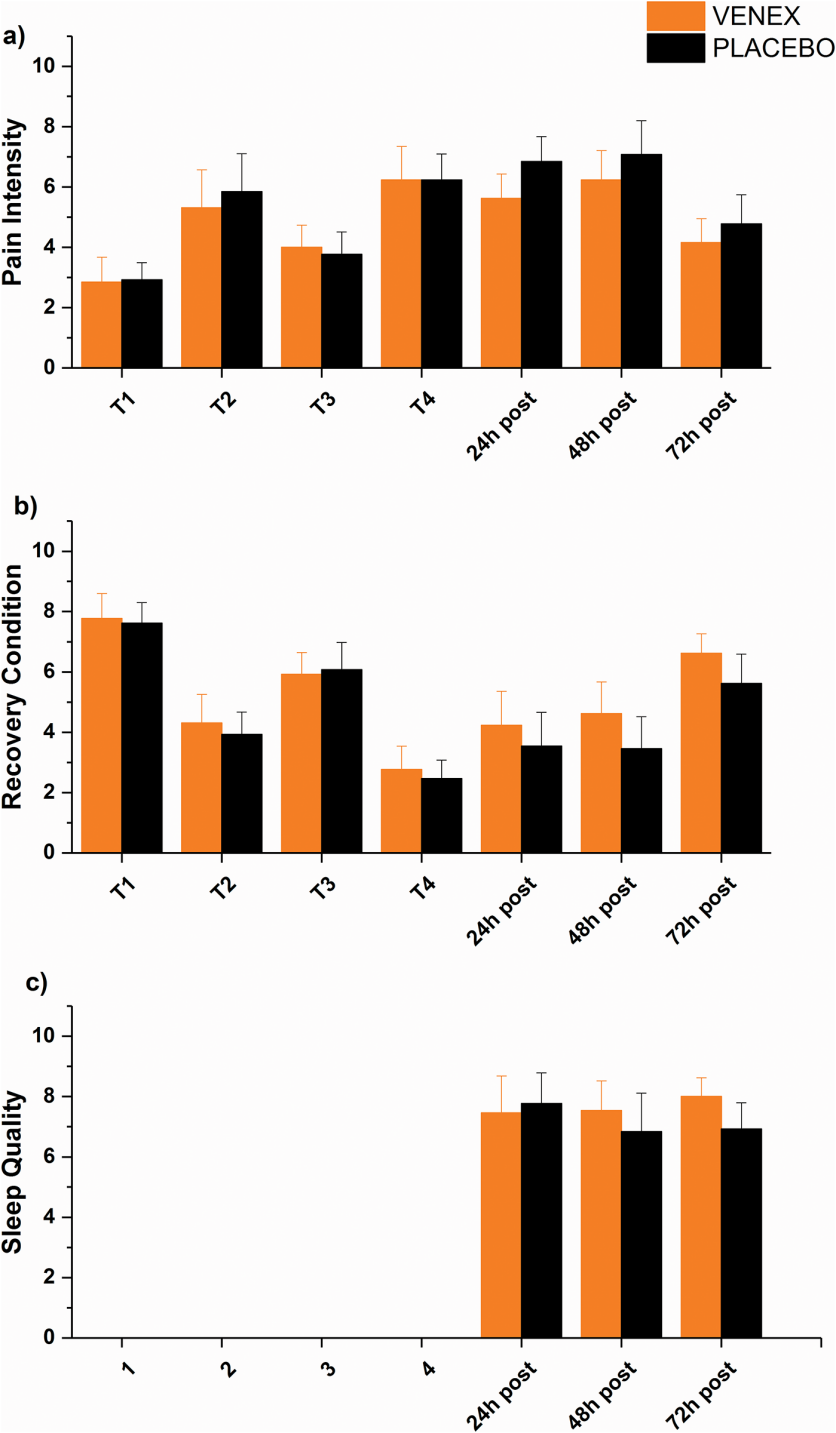
Mean ± 95% confidence interval of a) pain intensity (PI), b) recovery condition (RC) and c) sleep quality (SQ). There were no differences between treatments but a strong Cohen’s *d* of 0.8 for PI (a) 24h post and a medium Cohen’s *d* for RC (b) 24h post (0.6) and 48h post (0.7). Sleep quality (c) indicated a trend to significance with p = 0.068 during the third night and also a high Cohen’s *d* of 0.8 at this measurement.

No subjective parameter showed a significant difference for baseline. No significance difference was shown between treatments in the following measures but a strong between subjects Cohen’s *d* of 0.8 for pain intensity 24h post and moderate Cohen’s *d* of 0.6 and 0.7 for recovery condition 24h and 48h post in favour for Venex, respectively. Sleep quality showed a moderate non-significant effect (p = 0.068) with a high Cohen’s *d* of 0.8 in favour for the Venex treatment for the third night (Fig 4). Sleep duration showed no significant difference between the two treatments (p < 0.05). Pain intensity and recovery condition demonstrated significant differences (p < 0.05) over time as shown in Table 1. Mean data with standard deviation for all subjective parameters are shown in Fig 4 and Table 1.

## Discussion

The aim of this study was to investigate potential effects of Venex regeneration wear on neuromuscular, systemic and subjective parameters. Therefore, participants had to perform two exercise sessions on a single day in a randomized, single-blinded crossover design in order to track the recovery process when wearing Venex or a placebo product, respectively. Although our hypotheses could not be confirmed for neuromuscular and systemic parameters, the results of the subjective parameters indicated that the regeneration wear might have positive effects on subjective recovery. On a neuromuscular basis the hypothesis that parameters decrease less during the treatment with the Venex textile could not be verified for the MVC or the potentiated twitch torque. In combination with the similar voluntary activation (VA) observed during both treatments peripheral and/or central fatigue were not reduced during the Venex textile treatment.

Because of that we can state that our findings are not in line with results from Hasegawa and Katano (2014) [27] who found faster times for a curved sprint test after a second training session that was accompanied by an increased parasympathetic activity for the experimental group using the Venex textile. Because a MVC or potentiated twitch torque measurement is a much more specific test in terms of muscular fatigue than a curved sprint test we expected a faster recovery of both these parameters for the Venex treatment. One reason for the absence of enhanced muscular recovery with Venex could be the duration of wearing of the regeneration wear during the three hours rest between testing session two and three. On the other hand, even after 24h and a full night of wearing the garment our hypothesis that parameters return to baseline significantly earlier when participants wear the Venex textile could not be verified for any neuromuscular parameter.

Although the CMJ showed to be a useful test for explosive leg strength in terms of monitoring the recovery process [38] our results showed no significant difference between treatments. This again is in contrast to the findings of Hasegawa and Katano [27] but in line with our findings for MVC and potentiated twitch torque.

As a valid blood parameter for strength-training dependent fatigue creatinkinase (CK) seems to be the most striking parameter [39]. Based on the effects of the harmonized fiber (DPV576-C), which is supposed to provide a secretion of nerve growth factor (NGF) and thus an enhanced regulation of inflammation and an increased blood flow [15], CK would be expected to be eliminated earlier during the Venex compared to the placebo treatment. However, according to the CK measurements this assumption could not be verified. Only the single participant that was excluded from statistics for CK measurements showed a peak CK of 338 U·L^-1^ at T4 during the Venex textile treatment (434 U·L^-1^ during placebo at T4) and a decrease to 163 U·L^-1^ during Venex at T7 compared to an increase of up to >20000 U·L^-1^ for the placebo product. Because of no recognized methodical measurement errors, it is either possible that the washout phase was not long enough for this participant, that the participant responded extraordinarily to the Venex textile or that something out of the notice from the supervisor and participant happened during the treatment with the Placebo product. Additionally, and similar to CK, the subjective perceived pain of this participant showed strong differences in favor for the Venex textile, especially the second and third night after the exercise day (Venex: 6 (T5), 4 (T6), 2 (T7) versus Placebo: 8 (T5), 10 (T6), 8 (T7). The repeated bout effect could be another explanation for the different CK secretion although this participant had seven weeks of washout in between the two treatments (first treatment was with the placebo product) which should be a sufficient duration to eliminate effects of the repeated bout [29,30]. For this participant the findings of the neuromuscular measures are also in line with the findings of the biochemical and subjective measures. During the Venex treatment, MVC and twitch torque fully recovered at T6 while they were still strongly reduced during the placebo treatment (56.4% MVC and 66.6% twitch torque), but jump parameters (CMJ and RSP) were not affected. However, in order to not confound the results from this outlier data, this participant was excluded from statistics for the creatinkinase-activity.

It is important to state that the main performance parameters (MVC, CMJ and DJ) showed no significant differences between treatments. Accordingly, the question if athletes would tolerate higher training loads if they wear the Venex textile in between training sessions needs further investigation.

Based on the strong effect sizes for perceived pain and also recovery condition it can be stated that participants felt better even when the findings were not significant. This result would be in line with the excepted downregulation of TRPV4 and the increased secretion of NGF and thus a refined regulation of perceived pain. Further it is worth mentioning that most participants reported a notably heat when they wore the Venex textile, that however, was not evaluated on a scientific level. Nevertheless, the sensation of heat is in line with the suggested effects triggered by the far-infrared radiation of the Venex textile, as it might indicate an increased blood circulation [15].

Noteworthy, this study has some limitations. First, questionnaires to assess the status of recovery as well as the quality of sleep were not validated, but however, similar to a well-established pain scale [36]. Second, the sleep behavior was not tracked on a highly scientific basis since this was out of the scope of this study. However, further research on this issue is needed. Another limitation is the impact of nutrition on recovery. Although nutrition was documented, and the participants were requested to diet the same way as they did in the first treatment, this factor was not completely controlled.

Despite these limitations and no confirmation of the hypotheses associated with the suggested effects of Venex, the subjective parameters tentatively indicate that the organism reacts to the textile at least to a minor degree. Additionally, because sleep quality showed a trend to significance and a strong effect size for the third night it is speculated that the treatment with the platinum harmonized textile needs a longer duration for potential major and significant influences on the performance parameters as well as systemic parameters like CK. For further research on possible effects of platinum harmonized technology on human performance it seems reasonable to perform a longitudinal training study where participants have to wear the textile that is provided with the platinum harmonized technology in comparison to a placebo product and/or control. During such a study it might be helpful to track additional parameters like parasympathetic activity, resting heart rate, skin and core temperature, heart rate variability and/or blood flow when wearing Venex regeneration wear compared to a control and/or placebo in order to better understand potential effects of the regeneration wear.

## Supporting information

S1 File Survey questions English. [Survey questions English.docx]
(DOCX)

S2 File Survey questions original language (German). [Survey questions original language (German).docx]
(DOCX)
