# Correction: Influence of platinum harmonized textile on neuromuscular, systemic and subjective recovery

**DOI:** 10.1371/journal.pone.0191057

**Published:** 2018-01-09

**Authors:** Fridolin Zinke, Patrick Bakenecker, Daniel Hahn

Fig 2 is incorrect. The confidence intervals are missing. The authors have provided a corrected version here.

**Fig 2:**
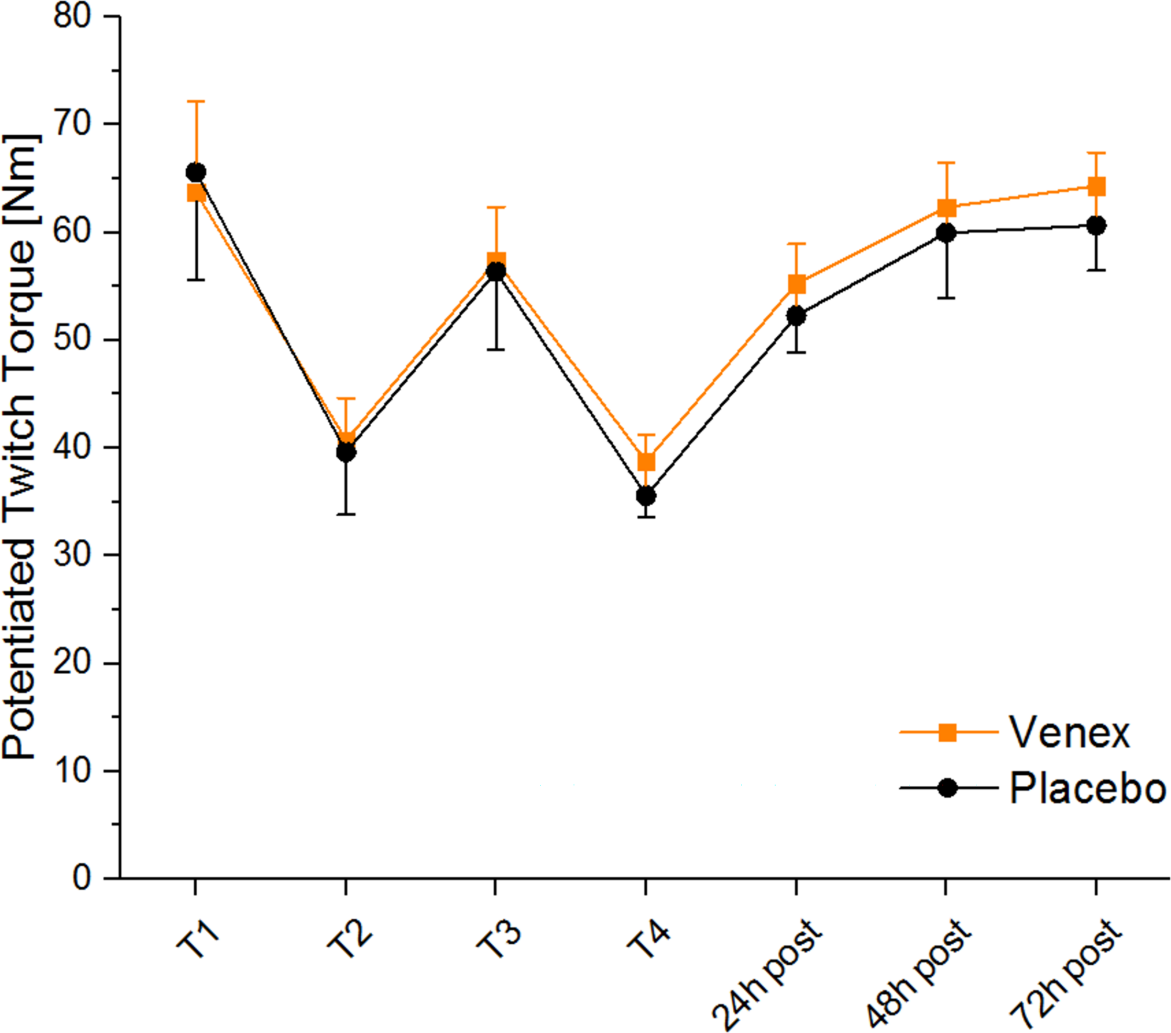
Potentiated twitch torque for both treatments over time. Data represent the mean ± 95% confidence intervals of the potentiated twitch torques.
