# Supplementary material for: Influence of platinum harmonized textile on neuromuscular, systemic and subjective recovery
Source: PLoS One. 2017 Oct 12;12(10):e0186162. doi: 10.1371/journal.pone.0186162 (PMC5638408; doi:10.1371/journal.pone.0186162)
Supplement: S1 File — [Survey questions English.docx] (DOCX) [file pone.0186162.s001.docx]

Participant:

Testing Session:

Recovery Condition:

1 2 3 4 5 6 7 8 9 10

Not recovered ------------------------------------------------------Fully recovered

Pain Intensity:

1 2 3 4 5 6 7 8 9 10

No pain -------------------------------------------------------------- Maximum pain

Slepp quality:

1 2 3 4 5 6 7 8 9 10

Bad -----------------------------------------------------------------------------Good

Sleep duration in hours:________

CMJ

1 2 3

DJ Height

1 2 3

DJ contact time

1 2 3
