# Supplementary material for: Influence of platinum harmonized textile on neuromuscular, systemic and subjective recovery
Source: PLoS One. 2017 Oct 12;12(10):e0186162. doi: 10.1371/journal.pone.0186162 (PMC5638408; doi:10.1371/journal.pone.0186162)
Supplement: S2 File — [Survey questions original language (German).docx] (DOCX) [file pone.0186162.s002.docx]

Proband:

Messzeitpunkt:

Allgemeiner Regenerationszustand:

1 2 3 4 5 6 7 8 9 10

Minimal regeneriert ---------------------------------------------Maximal regeneriert

Allgemeiner Schmerzzustand:

1 2 3 4 5 6 7 8 9 10

Minimaler Schmerzzustand ---------------------------- Maximaler Schmerzzustand

Schlafqualität:

1 2 3 4 5 6 7 8 9 10

Schlecht -------------------------------------------------------------------------Gut

Schlafdauer in Std.________

CMJ

1 2 3

DJ Höhe

1 2 3

DJ Kontaktzeit

1 2 3
